# Psychosocial Well-Being in Persons with Aphasia Participating in a Nursing Intervention after Stroke

**DOI:** 10.1155/2012/568242

**Published:** 2012-07-22

**Authors:** Berit Arnesveen Bronken, Marit Kirkevold, Randi Martinsen, Torgeir Bruun Wyller, Kari Kvigne

**Affiliations:** ^1^Department of Nursing Science, Faculty of Medicine, Institute of Health and Society, University of Oslo, P.O. Box 1130 Blindern, 0318 Oslo, Norway; ^2^Department of Nursing and Mental Health, Faculty of Public Health, Hedmark University College, P.O. Box 400, 2418 Elverum, Norway; ^3^Institute of Public Health, University of Århus, Nordre Ringgade 1, 8000 Århus C, Denmark; ^4^Faculty of Medicine, Institute of Clinical Medicine, University of Oslo, P.O. Box 1171 Blindern, 0318 Oslo, Norway; ^5^Department of Geriatric Medicine, Oslo University Hospital, P.O. Box 4956 Nydalen, 0424 Oslo, Norway

## Abstract

The psychosocial adjustment process after stroke is complicated and protracted. The language is the most important tool for making sense of experiences and for human interplay, making persons with aphasia especially prone to psychosocial problems. Persons with aphasia are systematically excluded from research projects due to methodological challenges. This study explored how seven persons with aphasia experienced participating in a complex nursing intervention aimed at supporting the psychosocial adjustment process and promoting psychosocial well-being. The intervention was organized as an individual, dialogue-based collaboration process based upon ideas from “Guided self-determination.” The content addressed psychosocial issues as mood, social relationships, meaningful activities, identity, and body changes. Principles from “Supported conversation for adults with aphasia” were used to facilitate the conversations. The data were obtained by participant observation during the intervention, qualitative interviews 2 weeks, 6 months, and 12 months after the intervention and by standardized clinical instruments prior to the intervention and at 2 weeks and 12 months after the intervention. Assistance in narrating about themselves and their experiences with illness, psychological support and motivation to move on during the difficult adjustment process, and exchange of knowledge and information were experienced as beneficial and important by the participants in this study.

## 1. Introduction

Aphasia, an acquired language disorder caused by brain damage, affects about one-third of the stroke population [1, 2]. About 40% continues to have significant language impairment at 18 months after stroke [3]. Aphasia ranges from mild, involving difficulties in finding words, to severe, involving severe impairment of all language modalities and leading to problems with expressions and comprehension of speech, reading, and writing and the use of language as a flexible tool in everyday life [4]. Language is the most important tool for human interplay, social participation, and community, and we make sense of life events and experiences through language [5].

The sudden and dramatic onset of aphasia following stroke is associated with major disruptions of everyday life and affects all dimensions of quality of life [4, 6]. The psychosocial adjustment process is complicated and protracted [7], and persons with aphasia (PWA) are especially prone to psychosocial problems, such as anxiety and depression [4, 8, 9], threatened identity [10], changes in their relationships with their significant others [11, 12], reduced social networks and social isolation/exclusion [13], unemployment, and abandonment of leisure activities [14]. The emotional and psychosocial factors have a marked impact on recovery, the psychosocial adjustment process, and the response to rehabilitation [4, 15, 16]. 

Several studies have sought to prevent and treat psychosocial problems in stroke survivors in general [17–20], but the outcomes differ substantially and the theoretical foundations of the effective components are unclear [21, 22]. However emotional support, information, and practical assistance are documented as important [21]. Two randomized controlled studies [23, 24] demonstrated that a systematic followup of stroke survivors with counseling [23] and “motivational interviewing” [24] significantly improved mood during the first year after a stroke. PWA were not focused on explicitly in these studies, and in the Watkins study, persons with severe communication problems were excluded. 

Interventions that include PWA pose methodological challenges due to the communicative barriers. This has resulted in a systematic exclusion of this target group outside the field of speech-and-language therapy [25, 26]. In a meta-synthesis of 293 qualitative research reports concerning chronic illness, Paterson, Thorne, Canam, and Jillings found that only two studies involved informants with impaired verbal communication [27]. In a systematic review of nursing rehabilitation of stroke patients with aphasia [28], the authors reviewed 24 papers relevant to nursing-specific interventions. Their key finding was that the integration of speech-and-language interventions and functional communication training into daily care could improve the effectiveness of speech-and-language therapy. The major focus was on functional communication training and screening tools to detect aphasia. In contrast, Hjelmblink and colleagues [29] found that the defined language impairment misled both the health care professionals and the PWA to focus only on language therapy, hence leaving the participant unsupported in other important aspects of rehabilitation. 

Nurses have a key role in stroke rehabilitation [24, 30, 31], and they have a unique possibility to follow patients over time and thereby secure continuity. We believe that nursing interventions tailored to assist PWA and their relatives in coping with psychosocial consequences of the stroke need to be targeted explicitly. In response to this, we developed a complex clinical intervention [32] aimed at supporting the psychosocial adjustment process and promoting psychosocial well-being the first year after stroke [33]. In this paper we focus on the experiences of the participants with aphasia which were included in the study. The aims of this study were to explore how the participants with aphasia experienced participating in the intervention and its impact on their recovery process and psychosocial well-being during and after the intervention.

## 2. Materials and Methods

### 2.1. Design

The current study was part of a larger study comprising 25 cases of stroke survivors followed during the first year after stroke. The larger study had a development and evaluation design guided by the UK Medical Research Council Framework [32], which is a recommended framework for developing and evaluating complex clinical interventions in health care. The development of the current theoretically and empirically informed intervention is reported in depth elsewhere [33]. In seven of the cases the participants had moderate-to-severe aphasia. These cases are explored further in depth in this paper. A qualitative multiple case approach was seen to be appropriate to explore the context-sensitive and multidimensional aspects of the individual recovery process such as coping resources, social network, age, gender, culture, and meaning attached to individual values and life concerns and how the intervention interacted with the individual challenges and needs [34]. 

### 2.2. Description of the Intervention

The recovery process following a stroke has been referred to as a demanding journey in which the stroke survivor moves through different phases as various challenges unfold [35, 36]. We used a metaphor translated to “The great trial of strength,” which refers to a prestigious and demanding bicycle race in Norway (lasting 15–20 hours), known by most Norwegians. Healthy and well-trained bicycle riders are followed by an escort car, which provides the support and equipment that the riders need. We assumed that a stroke survivor also needed an “escort car” to address different needs as they arose during the “recovery journey” after the stroke. Each session was conceptualized as a “pit stop” in this unpredictable “race” toward recovery.

The intervention was organized as an individual, dialogue-based, collaboration and problem-solving process between the stroke survivor and a trained nurse. Ideas from the approach “Guided self-determination” [37] were applied to assist the participants in developing new life skills to cope with psychosocial consequences of the stroke. The intervention addressed four dimensions of psychosocial well-being: Basic emotional state, meaningful activities, social relationships and self-concept [38], all of which are threatened by stroke [7, 14, 39, 40]. For each encounter we constructed worksheets addressing topics described as challenging in the stroke literature (related to the aforementioned dimensions of psychosocial well-being). The worksheets were adjusted linguistically for PWA. We used principles from the method “Supported conversations for adults with aphasia” [41] to facilitate the conversations. The intervention was planned to consist of eight encounters, each lasting about 1 hour, to be conducted during the first 6 months after a stroke.  Table 1 shows an overview of the guiding topical outline of the encounters and the topics of the associated worksheets which were planned for each encounter. Further information is available upon request.

### 2.3. Inclusion Criteria and Presentation of the Participants

The study was conducted in Norway in the period 2007–2009.

Inclusion criteria were adult persons (18 years or above) having suffered a stroke during the previous twelve weeks, medically stable, with sufficient cognitive functioning to participate (assessed by their physician/stroke team), interested in participating, and Norwegian speaking. Persons diagnosed with aphasia were broadly included due to the exploratory design.

Seven persons, one woman and six men, with moderate-to-severe aphasia were included. Their ages ranged from 33 to 72 years. A speech-and-language therapist assessed their language impairment and described their ability to produce speech, comprehend language, read, and write prior to the intervention. The participants entered the intervention program 4 to 12 weeks after stroke onset.  Table 2 gives a brief presentation of the participants in terms of medical and demographic data (prior to the intervention).

### 2.4. Duration and Context of the Intervention

The frequency of the encounters and the duration of the intervention period were initially planned to be eight encounters over 6 months. Among the participants with aphasia we had to prolong the intervention to complete the program because the participants needed more time, the trajectories differed, and medical complications necessitated adjustments. During a period lasting from 9.5 to 14.5 months, 10 to 16 encounters were carried out in each case, lasting from 40 minutes to 2 hours. The encounters took place in various locations depending on where the participants were in their trajectories, including the hospital, the rehabilitation unit, and the home. 

### 2.5. Methodological Consideration and Data Collection

Traditional methods for gaining self-reported data, such as interviews and questionnaires, are less suitable for PWA because of the presence of language impairment. Linguistic data from interviews are affected by changes in the syntactic, semantic, and pragmatic use of the language [13, 42, 43]. Questionnaires can also pose problems that result from the presence of nonlinguistic cognitive symptoms, such as apraxia, neglect, visuospatial problems [44], paresis, and vision disturbances [45]. In an attempt to meet these challenges, we assumed that triangulating various approaches would provide us with a more nuanced and complete picture of the experiences, given the limited ability of the participants to produce rich interview text [46]. Triangulation is recommended by several authors to improve trustworthiness of case studies [34, 47, 48]. The data were obtained by the following methods and data sources. 

#### 2.5.1. Participant Observation

We used participant observation during the intervention to achieve an insider's view of the process. In total, 89 individual encounters between each of the participants and the same intervention nurse were completed. The nurse wrote down systematic notes (referred to as log notes) after each session. The log notes described content of the dialogues, activity and interplay, the use of worksheets and communication resources, context, duration, and reflections. 

#### 2.5.2. Qualitative Thematic Interviews

The participants took part in three individual qualitative followup interviews at 2 weeks, 6 months, and 12 months after the intervention had ended (21 in total). The interviews were recorded on video to extend the limited verbal expressions with nonverbal data. The first interview focused on the experiences of participating in the intervention program and the perceived impact of the program on psychosocial well-being (i.e., mood, relationships, activity, and self-esteem). The second and the third interview were followup interviews based on the same themes and relevant topics that had been discussed during the intervention and in the previous interview(s). The interviews took place mainly in the participants' home (15), but some of them were conducted in a conference room at the hospital (5) and a rehabilitation unit (1). The interviews lasted from 50 minutes to 2 hours.

#### 2.5.3. Standardized Clinical Instruments

We applied four standardized clinical instruments addressing different aspects of subjective psychosocial well-being. The Stroke and Aphasia Quality of Life (SAQOL-39) is a disease-specific quality-of-life scale that measures a stroke's impact on the “physical”, “psychosocial,” “communication,” and “energy” domains [49]. Cantril's Ladder Scale more globally addresses satisfaction with life [50]. The Faces Scale measures the affective experience of happiness/sadness [51], whereas the Hopkins Symptom Check List (short version, HSCL-8) addresses symptoms of depression and anxiety [52]. All instruments were deemed to be appropriate for PWA because of the use of visuals [50, 51] and adjusted and simple text [52, 53]. The instruments were used three times: prior to the intervention (T1), two weeks after the intervention (T2), and one year after the intervention (T3). The instruments are further described in Table 3.

### 2.6. Data Analysis

The qualitative data was analyzed from a hermeneutic-phenomenological approach inspired by Ricoeur and Kvale [55–61]. This approach aims to understand the meaning of lived experiences through the interpretation of text. The text included verbatim transcripts of interviews, written expressions on the worksheets, drawings, images, pictograms, and log notes. According to Ricoeur [56], the metaphorical process plays a semantic role and contributes value to the meaning of a text through its ability to stimulate imagination and emotion. In this study the use of metaphorical thinking supported our interpretation and understanding throughout the analytical process, especially when the participants used single syllables, few words, drawings, artwork, nonverbal communication, or exchanged words.

The analytical process encompassed three main phases: naive reading, structural analysis, and comprehensive understanding [62]. In the first phase, the entire text from all cases was read to provide an overall impression. In the structural phase, each case was explored in depth, and each data source was analyzed separately as described below.

The interviews that had been recorded on video were viewed and transcribed verbatim. The transcripts were read separately and together with the video records several times to confirm the meaning of the text. The video supplemented the text with nonverbal expressions which were important to provide a richer interview text. Meaning units related to the aims of the study were identified and structured into themes. The log notes from all the encounters were further explored to extend the meaning of the themes that emerged from the interviews. For example, when the participants referred to events and experiences from the intervention, the log notes provided more detailed information about the content, the circumstances, and the actions associated with these sessions as well as where and when they took place.

Data obtained from the standardized clinical instruments were organized with a computer software program (PASW Statistics 18) to create graphical plot diagrams from all instruments to explore changes in the participants' statements over time. The Faces Scale and the Cantril's Ladder Scale are visual scales without verbal labels. We gave each face in the Faces Scale and each step in the Cantril's Ladder Scale numerical values to create graphical illustrations by the use of the computer software program. Secondly, the statements were interpreted in relation to the analyses of the qualitative data, ending in a summary (case record) for each case [47]. The data from the interviews, log notes, and instruments complemented each other. 

In the last phase, we compared the similarities and differences as well as the patterns across the cases in relation to the purpose of the study. Four of the authors (Berit Bronken, Marit Kirkevold, Randi Martinsen, and Kari Kvigne) conducted the analysis. Disagreements were discussed and led to further analysis, which was completed when the findings were redefined or confirmed. 

### 2.7. Ethical Considerations

A language therapist or a specially trained nurse outside the research group had obtained written informed consent from the participants before the study began. Oral and written information was communicated through the use of adjusted language and illustrated communication resources. A separate written informed consent was obtained before the postintervention followup interviews, that were recorded on video. The project was approved by The Regional Committee for Medical and Health Research Ethics and The Norwegian Social Science Data Services.

PWA are vulnerable to harm because of their reduced ability to express their meanings and reservations [63]. Autonomy, self-determination, respect, and ethical sensitivity were emphasized at all stages of the research process, considering the asymmetric relationship between the participants and the intervention nurse with regard to role, knowledge, language, and health condition. During the intervention, clinical care and security had priority over research goals. There was no conflict between the therapeutic goals and research goals. Small samples could compromise confidentiality [64]. To counteract this possibility, we have modified descriptions that might identify the participants. 

## 3. The Participants' Experiences of ****Participating in the Intervention and Its ****Perceived Impact on Their Recovery Process

Our findings are presented in two main sections; 3 and 4. First we present the participants' experiences of participating in the intervention and its perceived impact on their recovery process (3). In Section 4 we describe how the participants expressed their psychosocial well-being before, during, and after the intervention.

### 3.1. Assistance to Narrate about Themselves and Their Experiences

The participants expressed a great need to talk about events and experiences in their new life situation and about unfamiliar reactions and symptoms caused by the stroke. The long-lasting partnership made it possible to gradually coconstruct and frame their stories, which could be shared and further developed and discussed over time. The topics introduced by the worksheets were experienced as useful starting points for the subsequent conversations and stories. The stories were gradually constructed through an interactive process of talking, writing, using nonverbal expressions, drawings, and communication resources during the intervention (“toolbox” with illustrated materials). The stories were written down after each encounter (by the intervention nurse) and followed up in later encounters depending on the participant's individual wishes and needs. The interactive process of coconstructing stories is described elsewhere [65]. The content of the stories varied considerably and mirrored the individual life situation, needs, and goals of each participant. Individualization and flexibility were mentioned as important elements of the intervention, which was expressed like: “I felt free to decide myself what to talk about, no pressure or anything like that.”

All of them appreciated to tell who they used to be before they were hit by the stroke and how they struggled to adjust to their compromised roles as a partner, father, grandfather, friend, and worker after the stroke. They were all concerned about being perceived as competent adult persons, but difficulties in expressing themselves led to frustration and misunderstanding both in themselves and others in their close network. Facilitation to construct stories about events and experiences, receiving response, and discussing coping strategies were expressed as beneficial. The following quotes represent the sentiment among the participants: (The encounters) “lifted me up” and “raised me up.” The topics of the worksheets were experienced as relevant and important to talk about and described as beneficial in terms of “something concrete to focus on,” “interesting,” “systematic,” “awareness-raising,” and “helped to structure thoughts and feelings.”

For several of the participants, “the great trial of strength” metaphor, which was applied throughout the intervention, was experienced as a meaningful figurative representation of the recovery process and a beneficial “tool” to use when communicating about the ups and downs of recovery, energy, endurance, the physical challenge of negotiating difficult environments, meeting needs, and performing movements. One participant expressed it in this way:

*The metaphor has been meaningful because it [the trajectory] has been a great trial of strength. Now I am not in Oslo, and I am quite tired. If I had to cycle back, I would be very down [depressed. Pointing down to the ground]. One time [one stroke] is enough [smiling ironic]. I am at Lillehammer [half way of the race], and I still need one more year before I am back home again [healthy].*

#### 3.1.1. Reserved Time for Talking (Provided Opportunities to Talk)

Time to talk and give attention to psychosocial consequences of the stroke and aphasia was underscored as important to the participants. Most of them lacked conversation partners in their natural environment who were able to understand their situation and condition. The language impairment, as well as the emotional and cognitive symptoms, the tiredness, and the bodily experiences were difficult to explain to others and difficult for others to understand, which can be exemplified by this quote:

*We talk very little, very little... I think they [family and others] believe that there is something wrong with me, and that hurts me a lot... It is difficult for me to initiate a conversation and know what to talk about, which is difficult for others to understand.*

Several participants mentioned difficulties with initiating a conversation, not knowing what to talk about or how to express themselves. By specifically designating a time to discuss their psychosocial challenges, the participants were encouraged to tell about their daily experiences and life concerns, as expressed in this quote: “With the physiotherapist, I exercise; with the speech- and language therapist, I learn to talk, read and write. Here, I talk about me and my life.” The participant drew a picture of a sun and pointed to the rays, each representing a significant person or source of support. Another explained it in his way: “On one hand, it is difficult to walk. On the other hand, it is difficult to talk. I can walk or talk not both things at the same time. Time to talk is important!” He struggled immensely and took time to explain and underscore these important points. 

#### 3.1.2. Confidence to Talk

Several of the participants expressed embarrassment about their language disability outside of “treatment sessions.” They were afraid of being regarded as incompetent, drunk, or childish. One participant expressed it in the following way: “To talk like a child, when you are an adult; it does something to you!” Some of the participants had, in addition to their aphasia, problems with their voices, articulation, and speech apraxia. The following situation from one of the interviews illustrates this:

*During one of the interviews, a friend came by to invite the participant for a walk. The participant stopped talking and changed to nonverbal communication. He smiled, pulled his lips together and pointed, first at the intervention nurse and then at the watch, to indicate that he had to come back later. After the friend had left, the nurse asked why he had stopped talking and just pointed to his friend. [The intervention nurse was quite surprised, as this participant talked quite well if he had time to express himself].*

This participant said that he often avoided talking because he was afraid of making a fool of himself. He had been judged as drunk several times, which he was upset and offended about. The first time, a taxi driver assumed that he was drunk when the stroke first occurred. Later, during a call from a business connection, the associate quickly ended the conversation by saying, “I will call you back when you are sober.” Loss of the language as a tool to maintain positions and roles as professionals, competent family members, or friends often resulted in avoiding “exposing” situations. The encounters during the intervention were experienced as a “safe” place to communicate experiences related to the illness and daily life. Confidence, acknowledgement, respect, and knowledge were described as important elements in the collaboration process. 

### 3.2. Psychological Support

#### 3.2.1. Expressions of Thoughts and Feelings

All participants included in this study were enrolled after experiencing their first stroke. The sudden change from being healthy to being seriously ill and unable to talk about their condition was experienced as very difficult. The disruption of a well-functioning life caused by the stroke resulted in a condition characterized by fear, uncertainty, despair, and frustration. The importance of having a person available to support them in expressing their thoughts and feelings and communicate about them was emphasized by all of the participants, expressed like: “It helped me to put words on some of my thoughts and feelings, which were the most difficult for me.” At times, the seriousness of their strained situation was precarious to some of them, as illustrated by this quote: “Without x [the intervention nurse] and y [the speech and language therapist], I would have ended up at z [a mental hospital]!” During the encounters the participants' fear of suffering another stroke was recurrent, expressed in phrases like: “I was afraid of exercising and walking outdoors alone,” “I had to check that I could move and talk several times during the night,” “I'm still alive,” and “[....], if I don't have another stroke.”

The discrepancy between the participants' increased needs to talk and their reduced abilities is illustrated by the following quotes:

*I didn't understand what it was. I never had reactions like this before, but I couldn't say it! My language! It's not my, my... [pointing at his head], my language! *

*I was in coma for two days. When I woke up I didn't understand anything, anything. But the worst of all,...I couldn't talk.... It was terrible, terrible!*

The participants expressed experiences of being locked in like: “I was completely alone” or “I was in a bubble, and in that bubble it was just me.” Assistance to verbalize experiences helped breaking through the wall of experienced loneliness. 

The participants appreciated having their reactions confirmed as “normal” by communicating about these topics during the encounters, which provided some confidence and order in an otherwise confusing situation. Taking part in the intervention helped them to handle affective and cognitive strain by communicating with a knowledgeable professional from the field of stroke and aphasia. The participants said that they felt understood in terms of their compromised language and their situation as a “stroke survivor with aphasia,” as expressed in the two following quotes: “x [the intervention nurse] understood my language. I think we understood each other... mutually. Very good!” and “x [the intervention nurse] knows what it can be like to have aphasia and stroke.”

#### 3.2.2. Motivation to Endure and Continue

The long-lasting difficulties and the uncertainty about the duration and outcome of rehabilitation threatened the participants' confidence and future prospects. They had concerns about how they should sustain themselves personally, perform their future roles, and manage economically. The encounters were something that the participants looked forward to as a place where they could share their experiences and receive encouragement and response to continue. Their experiences of the encounters were expressed through statements like: “Raised me up,” “lifted me up,” “helped us to endure,” and “inspired me to not give up.” A middle-aged man explained it in the following way: “It's easy to just stay on the sofa and go down [expressed by a downward spiral hand gesture toward the floor].” Another participant, a man with comprehensive physical disability, severe aphasia, and a complicated clinical trajectory, described the importance of his participation in the intervention with two words: “Alpha and omega.” His wife, who took part in nearly every session, included the following comment:

*Without these sessions, my husband would never have returned home. I didn't think it was possible! I didn't know anything about stroke and have learned a lot. X [the intervention nurse] provided a contrast to all of the negative information that we got from the health care system. She believed in improvement and helped us to endure. We looked forward to the visits.*

The participants experienced the feeling of talking, despite their aphasia. Even the participant with the most severe expressive disability responded that “talking” was what he appreciated the most during the intervention. The following quote represent a sentiment: “To talk is very important. You need that in a situation like this.”

### 3.3. Exchange of Knowledge and Information Based on Individual Experiences

The opportunity to gain knowledge was underscored by all of the participants as an important aspect of the sessions. The exchange of knowledge and information based on the participants' own experiences was viewed as a help for the participants' self-understanding of the situation and to learn about stroke, aphasia, common reactions and trajectories, their rights, where to get help, and how to utilize the available coping resources. Limitations in speaking, reading, and writing made it difficult for the participants to gain information and navigate the health care system on their own. The importance of receiving feedback from professionals was mentioned by a number of the participants, as illuminated by the following quote: “Talking with someone with knowledge about my experiences, discussing them and getting some suggestions were helpful for me.”

Two of the youngest participants explicitly valued their roles as “co-researchers”; they felt useful and hoped that their experiences would help others with aphasia and stroke. Generally, the participants experienced the intervention as an important supplement to the other services they received, and they recommended that this form of intervention be made available to others with aphasia in the future.

## 4. The Participants' Expressed Perception of Their Psychosocial Well-Being and Life ****Situation before, during, and after the ****Intervention

In this section, we present how the participants expressed their life situation both in terms of qualitative data collected from the interviews and log notes and from their self-reported statements expressed by the standardized clinical instruments. 

### 4.1. The Road Has Been Hard

The “road” (recovery process) had been hard, long, and demanding for all of the participants. The consequences of the stroke changed their lives, and several described a “totally new world,” which they aspired to adjust to. Language difficulties and their consequences, such as tiredness, social barriers, and uncertainty, remained challenging. The relationships within families had been changed, and, in several cases, friendships had been lost [66]. Some of the participants also related the changes in their social activities to personal reasons, such as feelings of isolation, difficulties engaging with others and participating in arguments and discussions, and difficulties associated with concentration, understanding, self-expression, and maintaining their earlier roles. The following quote illustrates a perceived experience of being met as different: “They [family, friends and employer] think I am sick, but I'm not. I'm 80% healthy. I have problems with my language.” 

### 4.2. I Am Doing Quite Well, but It Is a New World

At the end of the intervention, all but one of the participants described their life situation as quite well, even though the days fluctuated with ups and downs and there were still challenges in everyday life, as illuminated in the two following quotes.

* Now I'm doing relatively well, but it is the damn language. It bothers me all the time. It is another world than the one that I came from.*

*I have been sick for about one week now [flu], but otherwise I mostly think it is going quite well. The fact that I can walk [with a stick and a lot of struggle] and sit here and... [pointing at the video camera] is fantastic! To talk is very important!... The road has been long and hard, and I still need one more year to get healthy.*

One of the participants experienced a setback in his recovery caused by a frightening attack of epilepsy. Psychological distress and anxiety were reactivated, and he struggled with unwanted side effects of medication, which affected his entire life situation. 

### 4.3. The Participants' Self-Reported Statements on the Standardized Clinical Instruments

In Table 4, we present the participants' statements on three of the standardized instruments: Cantril's Ladder Scale, the Faces Scale, and the Hopkins Symptom Check List across the three times of data collection. The statements are presented in numericals.

**Figure 1:**
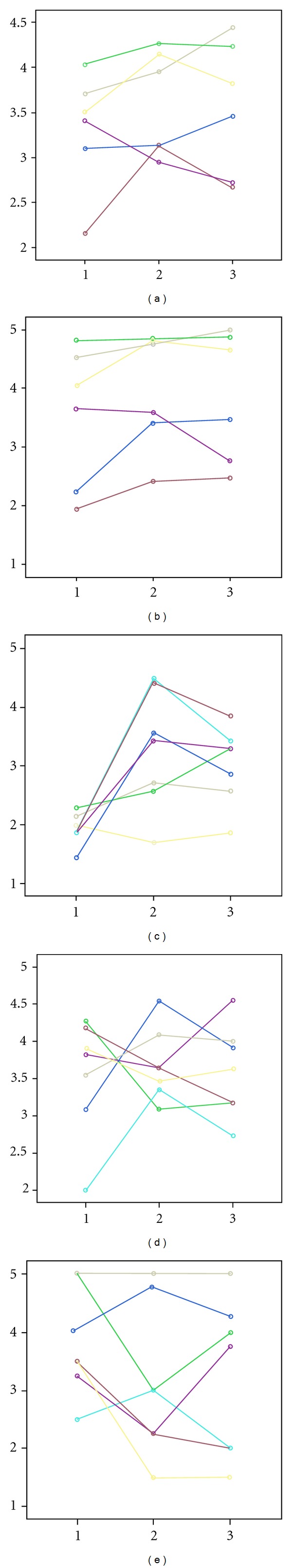
Plot diagrams of the participants' statements on SAQOL-39. SAQOL-39: Stroke and Aphasia Quality of Life Scale. Dimensions: (a) total score; (b) physical function; (c) communication; (d) psychosocial functioning, and (e) energy. *Value scores (y-*axis). Range score: 5-1. Score 5 is no trouble at all; score 4 is a little trouble; score 3 is some trouble; score 2 is a lot of trouble; score 1 is could not do it at all. *Time (x-*axis) Time 1: T1, before the intervention (5–12 weeks after stroke). Time 2: T2, two weeks after the intervention (about 1 year after stroke). Time 3: T3, 12 months after the intervention (about 2 years after stroke).

Figure 1 demonstrates changes over time on the SAQOL-39; total score (a), physical function (b), communication (c), psychosocial function (d), and energy (e).

**First Assessment (T1)**

At baseline, the participants self-rated their perceived global life satisfaction from middle to very good (Table 4, Cantril). Emotionally, all of the participants but one expressed themselves to be quite happy (Table 4, Faces), and they were slightly bothered by psychological distress (HSCL-8). There was great variation in the physical affection of the stroke. Two of the participants struggled significantly or were unable to perform the functions that the questions asked for. In one case, statements for the physical dimension of the SAQOL-39 are missing because there were several activities that this participant had not yet tried. All of the participants struggled a lot with communication, with scores between 1.25 and 2.25 (Figure 1(c)). The participants rated their psychosocial situations quite well (Figure 1(d)). In one case, the statements were lower than in the other cases. This man expressed himself as extremely sad and dissatisfied with his life situation (Table 4, Faces and Cantril).

**Second Assessment (T2)**

Two weeks after the intervention, about one year after the stroke, the participants' statements of their life satisfaction generally changed in a positive direction in four of the cases, were unchanged in two cases, and declined in one case compared with statements at T1 (Table 4, Cantril). Two participants stated themselves as happier at T2 than at T1, but in five of the cases the emotional statements tended to drop slightly. However none of them tended to be sad (Table 4, Faces). Five participants were slightly bothered with psychological distress, while two had bothersome symptoms; one participant was anxious and tired, and one was tired all the time (Table 4, HSCL-8). Changes expressed by the SAQOL-39 showed slight improvements in six of the cases and decline in one (Figure 1(a)). The degree of the relative changes in scores for the different subdimensions in each case varied substantially. Physical function improved slightly in most of the cases and markedly in one. Communication improved markedly in most of the cases, slightly in one and was almost unchanged in one (Figure 1(c)). Three participants showed psychosocial improvement, one remained relatively unchanged, and three declined (Figure 1(d)). Low energy and tiredness were marked in four of the cases, while the scores improved or remained unchanged in the three remaining cases (Figure 1(e)). In the case where the total score on the SAQOL-39 declined, the energy level dropped critically from 3.50 to 1.50, which probably explained the other scores (Figure 1(e)).

**Third Assessment (T3)**

At the long-term followup, one year after the intervention and about two years following the stroke, the participants' general satisfaction with life varied; two were very satisfied, but most of them reported that they were moderately satisfied. One had a score that was below the middle of the scale (Table 4, Cantril). Emotional well-being was generally stated as quite well; two were very happy, one was happy, three were somewhat happy, and one was neither happy nor unhappy (Table 4, Faces). In five of the cases, the total scores on the SAQOL-39 declined between T2 and T3, which may be explained by the surprising decline in ratings for communication in five of the cases and decline in psychosocial function in four cases (Figures 1(c) and 1(d)). Two participants reported that they continued to have some bothersome symptoms with tiredness and some psychological distress (Table 4, HSCL-8).

In summary, from baseline (T1) through the follow up, the participants' self-rated statements on the instruments varied. The total score measured by SAQOL-39 improved in six of the cases and declined in one. Tiredness and low energy explained most of the changes in this subject, which probably impacted the other scores. During the intervention, this participant was admitted to the hospital with suspicion of a new stroke, which delayed his recovery.

### 4.4. Integration of Data from the Interviews, the Log Notes, and the Instruments

The complicated recoveries of the participants were to some extent mirrored by the changes in their statements on the standardized clinical instruments, and the range of the statements (scores) was described and explained based on the qualitative data provided by the participants during the intervention. Within the first weeks following the stroke (T1), all but one of the participants were still inpatients at the hospital or in a rehabilitation unit, that is, in safe environments with access to professionals. They were happy about surviving and had an attitude that the extent of the stroke could have been worse (referring to others). They believed that their language difficulties would resolve after a period of active training. At this time, the participants were mainly optimistic, with the exception of one who experienced deep grief, not because of the stroke, but because a close friend had recently died from a serious illness.

The evolving consequences of the language disorder and the stroke became gradually clearer to them as the challenges of everyday life, family, work, and society arose after returning home. The long-lasting problems and the uncertainty about the outcomes of the rehabilitation were difficult to understand and handle.

In four of the cases, one or several medical complications negatively impacted the recovery processes; two participants experienced frightening attacks of epilepsy, three were readmitted to the hospital because of symptoms of a new stroke (confirmed in one case), and one fell and incurred a complicated hip fracture resulting in a prolonged trajectory.

Profound life events within the family and/or at work, such as illness of a close family member, partner crises, and unfair dismissal from work, occurred in three of the cases. For example, one participant was surprisingly fired from work before even being offered a chance to try (two weeks before measurement T2). In another case, the participant's colleagues noted and documented mistakes (behind his back) to legitimate a dismissal of him. All of these events were difficult to handle for people who lacked a “normal” ability to defend themselves on top of other challenges. Loss of energy was explained by the participants' continuous efforts to concentrate, understand, and express themselves and by externally induced energy loss caused by waiting, difficulties obtaining information from health and social services, and, in some cases, unclear messages from employers.

In summary, the information obtained from the various data sources complemented each other and provided a nuanced picture of the participants' experiences of their recovery process and their subjective psychosocial well-being during and after the intervention. The qualitative data provided detailed information about the meaning of the challenges and changes that the participants experienced over time, which also were expressed by the statements on the standardized clinical instruments. 

## 5. Discussion

To the best of our knowledge, there has been no similar psychosocial nursing intervention tailored to stroke survivors with aphasia. Participation in the intervention was experienced by the participants as an important source of support during the adjustment process. The intervention promoted their sense of psychosocial well-being through facilitating their expressions as they talked about themselves and their experiences in a new and changed life situation. The intervention also provided psychological help in terms of affective, cognitive, and motivational support during a demanding and hard “journey” of recovery. Exchange of knowledge and information was also emphasized as an important aspect of taking part in the intervention program. All these elements are documented as key elements for a successful recovery process in other studies as well [67, 68]. Our findings also correspond with findings from studies focusing on a life-coaching approach to aphasia, which highlight that learning to live well with aphasia takes time, aphasia concerns the whole family, and the goal is to help PWA to fit the consequences of stroke and aphasia into their changed lives [69].

The way the intervention was tailored and organized enhanced the participants' experiences of “talking” and sharing their stories. Storytelling is in general considered to be essential to self-understanding and sensemaking [5, 70, 71], and the act of narrating and sharing stories about experiences with illness is generally acknowledged [72] and considered as a valuable part of the recovery process after stroke [73–76]. For people with aphasia, the normally taken-for-granted ability of language is seriously disrupted [6] by their reduced ability to transform experiences, thoughts, and feelings into language and stories. The participants in the present study experienced fewer options for expressing themselves, being listened to, and being met with understanding than they were used to before stroke onset. According to Frank [77], stories are intentional, meaning that they are told with the intent that they will be listened to, and acted upon. PWA need support to tell their stories, be listened to, and interact socially.

The method “Supported conversation for adults with aphasia” points to the responsibility and the skills of the nonaphasic conversation partner to facilitate conversations with people with aphasia [41]. Clinical interventions that can support and promote the narrative processes of people with aphasia are repeatedly called for in other studies [78, 79]. Simmons-Mackie and her colleagues [80] found that partner training was effective in improving communication activities and/or participation when individuals with aphasia interact with trained communication partners. They highlighted the need for nurses to be particularly capable in communicating information about aphasia and its course to PWA and their families. The role of skilled nurses working with PWA is also highlighted in other studies [81–83]. Professional acknowledgement of unfamiliar symptoms and reactions, as well as further discussions about ways to handle them, was experienced as beneficial. The value of professional legitimization of common reactions to changes caused by chronic illness in general was outlined by Bury in 1982 [6].

The exchange of personal and professional knowledge based on the contextualized real life experiences of the participants formed the basis for the active collaborative partnership between the intervention nurse and the participants in our intervention. According to the theoretical foundation of this intervention [33], professional competence (nurse) and personal competence (participants) were regarded as different but equally important, and they were considered complementary sources of knowledge exchanged through dialogues and mutual active participation [37]. Ellis and colleagues [21] found that knowledge provision and teaching, combined with self-study, and individualized counseling, and support were more effective than passive approaches for important outcomes such as depression and anxiety. PWA were not mentioned explicitly in that review, but the findings appear to be transferable to this group and are in line with our approach.

The statements on the standardized instruments mirrored the real-life experiences of challenges and coping expressed in the qualitative data during and after the intervention. Support during the difficult process of adjusting to change was perceived as helpful and beneficial. Several of them expressed that they needed support to endure. Depression and psychological distress in PWA after stroke are common and vary over time [9, 84]. In Kauhanen's study [9], severe depression increased from 11% at three months to 33% at twelve months after stroke. We measured symptoms of psychological distress using the Hopkins Symptom Check List (HSCL-8). According to this instrument, only one participant reached the suggested cut-off score (at T3) used in other studies [54]. In our study findings both from the qualitative data and from statements on the instruments suggest that the participants are quite happy and relatively satisfied with life. We have reflected on whether participating in the intervention program contributed to the prevention of depression, but we cannot answer this question based on data from this small group. However, it would be of interest to evaluate this hypothesis in a larger, controlled study.

The changes in the scores on the SAQOL-39 (Figures 1(b) and 1(c)) tended to remain the same between T2 and T3, and for some items, there was a slight decrease. This might be associated with the absence of the “intervention sessions” during this period, as several of the participants lacked conversation partners with whom they could communicate well. Reduced levels of energy to maintain social connections also led to social isolation. Social support and interaction with others are acknowledged as essential for sensemaking, self-image, and identity adjustment [71], all of which are threatened in PWA [10, 85], and social support has been identified as a critical factor in living successfully with aphasia [67, 69, 86, 87]. Associations between lack of social support and psychological distress in PWA are also reported by others [84].

Adequate measurement and assessment of the outcomes of intervention studies is generally difficult [32] and probably even more challenging in studies including PWA [68, 88]. The triangulation of data from several sources in the present study helped us to understand the meaning behind the participants' expressions gathered by participant observation, interviews and clinical instruments. The reality of living with aphasia was still challenging, and the life situations of the participants were not ideal, but nonetheless, they were quite happy and satisfied. Participation in the intervention program was expressed as an important contribution to their psychosocial well-being during the first year after stroke. 

### 5.1. Strengths and Limitations

The longitudinal design, with data collection both during the intervention and during three followup sessions, using triangulation of data as well as the designated methods, is thought to extend our understanding and improve the validity of the findings [46, 47]. Findings from the log notes, the interviews, and the standardized clinical instruments provided us with a significant amount of data from a group with reduced production of speech.

The same nurse performed the encounters and the followup interviews, and we believe that this was the best way to maximize the information from the participants considering their individual ways of communicating and their vulnerable situation. Trust and confidence in each case was developed over time, and the longitudinal design contributed to a continuity which allowed for a better understanding of the participants' recovery process, and the development of increasing richness and depth of the data over time. Both active participant observation and interviews with PWA present particular risks related to the subjective selection of quotes because this group often needs support to initiate conversations and assistance in keeping conversations going. This might be a potential source of bias [61]. The participants confirmed that they had expressed what was important to them during the interviews, but some of them also commented that they not were able to express themselves in the way they wanted to. Videotapes of the postintervention followup interviews were valuable for the transcription of the interviews (total communication) and the analytical process. They also enhanced the transparency between the authors who took part in the reflexive and analytical process and analyzed the text for competing interpretations. During the intervention, however, we opted not to tape the encounters due to ethical and pragmatic reasons, as the participants were included in an early phase after stroke when they were still inpatient in acute care hospitals or rehabilitation units. Doing so would probably have enhanced the analysis of the field notes but would also have distracted the attention during the dialogues and increased the costs.

The participants in this study were younger than the average stroke survivor, which is about 76 years old (75.3 for men and 77.7 for women) in Norway [67], and only one woman participated. Generalization from this small group is not possible, and nor was it intended, but our findings correspond with comparable findings from other studies, and we believe that knowledge from our study can be transferable to others engaged in psychosocial rehabilitation to PWA following stroke. The participants also received therapy from other health care professionals, which they also underscored as important. More investigation is needed to explore the interplay between different services. 

## 6. Conclusion

Facilitating PWA with narration about themselves and their experiences, the provision of psychological support during a demanding recovery process, and the exchange of knowledge and information were experienced as beneficial to the recovery process in seven participants of a clinical intervention program. We believe it would be worthwhile to test this intervention in a larger context in future research.

## Figures and Tables

**Table 1:** Topical outline of the intervention (guiding structure).

Encounter	Aim	Worksheet
One	To establish a relationship for collaboration in an early phase after the stroke.	1a: Invitation to collaboration
		1b: The stroke—what happened?

Two	To gather knowledge about personal values, interests, and goals as a common platform for further collaboration. (Who are you (identity), which life is interrupted by the stroke?). To prepare for further collaboration after home coming.	2a: Life line—Personal background, values and interests 2b: Metaphor—“The great trial of strength”

Three	To support the participant in their process of adjusting to a changed situation “from healthy to stroke survivor in everyday life.” To support the participant in clarifying setting goals (short terms) and opportunities.	3a: Personal metaphor of your life as a stroke survivor 3b: Mood in everyday life (unfulfilled sentences)

Four	Invitation to narrate about bodily experiences and changes. Support in making sense of the new experiences and mobilize available recourses. Renegotiate new roles and identity adjustment.	4a: Me and my life (unfulfilled sentences) 4b: My body (graphical illustration of a woman/man)

Five	Identify goals to focus and sort out what has to be done by whom to reach the goals. Support to identify personal resources and significant resources in their network.	5a: Problem-solving process 5b: Daily activities in everyday life (unfulfilled sentences) 5c: Relationship with others (unfulfilled sentences)

Six	Help to integrate illness and life in a way that appear manageable for the participant. Support to promote health and build up resistance resources (i.e., sleep, social relationships, meaningful activities, food, physical activity). Support to develop new life skills that are necessary to live well with changes caused by the stroke.	6a: Illness and life 6b: Choice of aims to focus 6c: Balance in everyday life 2b: Metaphor (How is the terrain you are moving in now? What kind of support do you need from the “escort car”)

Seven	Talk about experiences and support the coping process. Assistance to be conscious about personal recourses and recourses in their network/environment.	7a: Coping in everyday life 7b: Habits I need to change/should change 7c: Network

Eight	Negotiating perspectives and goals for the further recovery process. Summarizing the collaboration process.	2b: Metaphor (past-present-future)

**Table 2:** Medical and demographic information describing the participants.

Participants	Physical extent of the stroke	Language ability	Civil status	Work
Man, 53 years	Hemorrhagia of the left hemisphere. Paresis in right side. Can walk. Visuospatial neglect.	Word production and understanding seriously affected, no functional reading or writing ability.	Lived together with wife and three children (teenagers).	Full time

Man, 72 years	Thrombosis of the left hemisphere. Hemiplegic right side. Paralysis in right arm. Not able to walk alone.	Speech production and speech understanding seriously affected. No functional reading or writing ability. Good situational understanding.	Lived together with wife. One grown child and one grandchild.	Retired

Man, 63 years	Thrombosis of the left hemisphere. No visible motor symptoms. Independent of help.	Speech production seriously affected. Speech apraxia. Disability in reading and writing. Good understanding.	Lived alone. Two grown children and one grandchild.	Full time

Man, 43 years	Thrombosis of the left hemisphere. Slight numbness and reduced strength in right side. Diplopia.	Expressive and impressive difficulties. Understanding better than production of speech. Reading and writing disability. Good situational understanding.	Lived partly alone. Two children (teenagers).	Full time

Man, 60 years	Thrombosis of the left hemisphere. Hemiplegic right side, some neglect. Can walk short distances with a stick.	Understanding is better than production of speech. Requires time to find words. Reading and writing disability.	Lived alone. Two grown up children and two grandchildren	Full time

Woman, 33 years	Thrombosis of the left hemisphere. Paresis in right side.	Serious expressive and impressive difficulties. Sound and word paraphasia. Strongly reduced reading and writing disability. Situational understanding is good.	Lived alone.	Full time

Man, 64 years	Hemorrhagia in the left hemisphere. Reduced strength in right side. Can walk with a roller. Reduced ADL function. Reduced vision. Concentration and memory affected.	Good speech production and understanding, reading and writing disability, dysarthria.	Lived in a nursing home after stroke onset. No children.	Disability benefit

**Table 3:** Standardized clinical instruments.

Type	Instrument	Concepts	Scores
			39 items.
Health-related quality of life	Stroke and Aphasia Quality of life SAQOL-39 [49]	Disease specific quality of life	Four dimensions rating the extent to which the informants struggle with different functions.
			Scoring: total score and four subscores (physical function, communication ability, psychosocial life, and energy). Range: 5–1, “no trouble at all” (5) to “could not do it at all” (1).

Global evaluation	Faces Scale [51]	Emotional well-being	Seven visual faces whose expressions vary from very happy to very sad. The scale does not have verbal labels, but each face was given numerical values for the purpose of graphical illustration. The most happy face was given the numerical value 7 and the most sad face was given the numerical value 1.

Global evaluation	Cantril's Ladder Scale [50]	Life satisfaction	Visual ladder with ten steps and 11 numbers ranging from 10 to 0: step ten at the top of the ladder depicts the highest level of satisfaction, and step one depicts the lowest. The scale does not have verbal labels, but was given numerical values for the purpose of graphical illustrations. Step ten was given the numerical value 10, step 1 the numerical value 0.

Symptom specific	Hopkins Symptom Check List—8 items [52, 54]	Psychological distress/mental health	Eight statements related to common symptoms of anxiety and depression with scores ranging from 4 to 1: “not bothered” (4) to “very bothered” (1).

**Table 4:** The participants' statements on the Cantril, Faces, and HSCL instruments.

Case	Cantril			Faces			HSCL-8		
	T1	T2	T3	T1	T2	T3	T1	T2	T3
1	7	9	9	5	7	5	3.25	4.00	3.75
2	7	8	9	7	4	7	4.00	3.75	3.88
3	7	7	6	7	4	7	4.00	3.86	3.75
4	8	9	6	7	6	5	3.75	2.00	3.13
5	8	6	6	6	5	6	3.88	3.75	3.63
6	6	6	5	5	4	4	3.50	3.25	2.75
7	3	5	4	1	4	5	3.63	3.75	3.86

**Cantril**: Cantril's Ladder Scale, life satisfaction (global).

Presents a picture of a ladder with 10 steps and 11 numbers (0–10).

Step ten at the top of the ladder depicts the highest level of satisfaction (10), and step one at the bottom depicts the lowest (0).

**Faces**: Faces Scale, affective experience of happiness/sadness.

Presents seven visual faces whose expressions vary from very happy (7) to very sad (1).

**HSCL-8**: Hopkins Symptom Check List with 8 items, symptoms of psychological distress (depression and anxiety). Range score: 4–1. Score 4 is not bothered, 3 is to a less degree bothered, 2 is quite bothered, and 1 is very bothered.

**Time**

T1: before the intervention (5–12 weeks after stroke).

T2: 2 weeks after the intervention (about 1 year after stroke).

T3: 12 months after the intervention (about 2 years after stroke).
